# FOXQ1 promotes pancreatic cancer cell proliferation, tumor stemness, invasion and metastasis through regulation of LDHA-mediated aerobic glycolysis

**DOI:** 10.1038/s41419-023-06207-y

**Published:** 2023-10-24

**Authors:** Changhao Wu, Chenglong Zheng, Shiyu Chen, Zhiwei He, Hao Hua, Chengyi Sun, Chao Yu

**Affiliations:** 1 Department of Hepatobiliary Surgery, The Affiliated Hospital of Guizhou Medical University, Guizhou Medical University, 550001 Guiyang, China; 2 College of Clinical Medicine, Guizhou Medical University, 550001 Guiyang, China; 3 Guizhou Provincial Institute of Hepatobiliary, Pancreatic and Splenic Diseases, 550001 Guiyang, China; 4 Key Laboratory of Liver, Gallbladder, Pancreas and Spleen of Guizhou Medical University, 550001 Guiyang, China; 5 Guizhou Provincial Clinical Medical Research Center of Hepatobiliary Surgery, 550004 Guiyang, Guizhou China; 6 Department of Hepatobiliary Surgery, Shenzhen Key Laboratory, Shenzhen University General Hospital, 518000 Shenzhen, China; 7 Department of Hepatic-Biliary-Pancreatic Surgery, South China Hospital, Medical School, Shenzhen University, 518116 Shenzhen, China

**Keywords:** Cancer metabolism, Cancer genomics, Cancer therapeutic resistance

## Abstract

Pancreatic cancer (PC), a gastrointestinal tract malignant tumor, has a poor prognosis due to early metastasis and limited response to chemotherapy. Therefore, identifying novel therapeutic approaches for PC is critical. Epithelial–mesenchymal transition (EMT) is known as the vital progress in PC development, we constructed the EMT-related prognosis model to screen out that FOXQ1 probably involving in the EMT regulation. FOXQ1 has been linked to the malignant process in a number of cancers. However, its function in PC is unknown. In our work, the expression of FOXQ1 was elevated in PC tissues, and a high level of FOXQ1 in PC was linked to patients’ poor prognosis. FOXQ1 overexpression promoted aerobic glycolysis and enhanced PC cell proliferation, tumor stemness, invasion, and metastasis. Whereas, FOXQ1 silencing showed the reverse effect. Furthermore, mechanistic studies indicated that FOXQ1 promotes LDHA transcription, and thus modulates aerobic glycolysis to enhance PC cell proliferation, tumor stemness, invasion, and metastasis by increasing LDHA expression. Therefore, these novel data suggest that FOXQ1 may be a possible therapeutic target in PC.

## Introduction

The incidence of pancreatic cancer (PC) has grown annually by 0.5–1.0% over the past two decades [1, 2]. PC is exceedingly aggressive and the 5-year survival rate is <10% [3]. The majority of patients already have distant metastasis at the time of diagnosis, making them ineligible for surgery. The mechanisms of distant metastasis in early-stage PC and cancer recurrence after treatment, which lead to poor prognosis, have been a hot topic of research [4]. Epithelial–mesenchymal transition (EMT) is a crucial mechanism in tumor cells that encourages early distant metastasis [5]. EMT influences PC resistance to chemotherapy and is closely linked to PC’s poor prognosis [6]. EMT-related targets for PC diagnosis and potential therapeutic strategies have not been identified and therefore, identifying new targets is critical to improve PC diagnosis, treatment efficacy, and patient prognosis [7].

The transcription factor forkhead box Q1 (FOXQ1), a member of the FOX family [8], contributes to a variety of physiological processes, including cellular senescence [9], glucose metabolism [10], lactate synthesis [11], and cardiac fibrosis [12]. FOXQ1 also plays a significant role in the biological processes that contribute to malignancy, including invasion, apoptosis, and EMT [13] in diverse cancers, including breast cancer, rectal cancer, and intrahepatic cholangiocarcinoma [14–18]. Nevertheless, the role of FOXQ1 in PC has not been clarified.

Growing evidence has indicated that reprogrammed metabolism may be crucial to PC development, progression, therapy, and prognosis [19]. Glycolysis is the primary mode of energy production that sustains cancer cell proliferation and metastasis, even under normoxia conditions [20]. Increased glycolysis is a common and essential feature of cancer metabolism, which is highly dependent on improperly functioning metabolic enzymes. Lactate dehydrogenase A (LDHA), which converts pyruvate to lactate, enhances the glycolytic process [21]. The expression of LDHA is unusually increased in numerous cancers and is linked to the development of malignancy [22]. Moreover, LDHA-mediated aerobic glycolysis influences the EMT process during carcinogenesis [23]. By reversing the “Warburg effect,” targeted LDH inhibition can stop the development of tumors [24]. LDHA is highly expressed in PC, suggesting that the development of LDH inhibitors may be a possible route for PC treatment [25].

In the current work, we explored the role and mechanism of EMT-related gene FOXQ1 in the proliferation, tumor stemness, invasion, and metastasis in PC. Our results showed that PC has elevated FOXQ1 expression, which was significantly related to a poor clinical outcome. We further found that FOXQ1 binds to the LDHA promoter and facilitates the transcription of LDHA. These activities enhanced aerobic glycolysis and LDHA expression, which promoted PC cell proliferation, tumor stemness, invasion, and metastasis. Our findings reveal the role and mechanism of FOXQ1 in driving PC development, suggesting FOXQ1 may be a possible treatment target for PC.

## Materials and methods

### Bioinformatics analysis

The EMT-related dataset (Supplementary Table 1) was retrieved from the GSEA database (https://www.gsea-msigdb.org/gsea/index.jsp). The PC RNA-seq data was downloaded from the TCGA database (https://portal.gdc.cancer.gov/). The TCGA and GTEx data were combined using GEPIA (http://gepia.cancer-pku.cn/index.html). The HumanTFDB website (http://bioinfo.life.hust.edu.cn/HumanTFDB) was used to predict transcription factor binding locations.

### Sample collection

Ninety-six pairs of PC and surrounding normal pancreatic tissues were obtained. The Ethics Committee of Guizhou Medical University approved the collection and use of clinical specimens, and all patients provided written consent.

### Immunohistochemistry (IHC)

Tissue slices were fixed, embedded, deparaffinized, and blocked. The sections were incubated with anti-FOXQ1 (Proteintech, Cat No. 23718-1-AP), anti-KI67 (Servicebio, Cat No. GB111499), anti-PCNA (Servicebio, Cat No. GB11010), anti-N-cadherin (Cell Signaling Technology, Cat No. 13116), anti-Vimentin (Signalway, Cat No. 33541), and anti-LDHA (Proteintech, Cat No. 66287-1-Ig) antibodies at 4 °C overnight. After washing with phosphate-buffered saline (PBS), the sections were incubated in a secondary antibody (Proteintech, Cat No. PR30009) for 1 h at room temperature. Hematoxylin re-staining, imaging, and DAB staining were then performed using standard procedures. Two different pathologists independently assessed the findings.

### RNA extraction and RT-qPCR

Total RNA was extracted using Trizol (Invitrogen, Cat. No. 10296010) and the concentration was measured with the Nano Drop ND1000. The *TransScript*^®^ Two-Step RT-PCR SuperMix (TransGen Biotech, Cat No. AT401) was used to generate cDNA, and *PerfectStart*^®^ Green qPCR SuperMix (TransGen Biotech, Cat No. AQ601) was used for RT-qPCR. Gene expression was calculated using the 2^−ΔΔCT^ method. The corresponding primer sequences are given in Supplementary Tables 2 and 3.

### Western blot

Lysis of cells was conducted with the use of RIPA buffer containing protease and phosphatase inhibitors on ice for 20 min (Thermo Fisher Scientific; Waltham, MA, USA). After centrifugation at 12,000 rpm for 15 min at 4 °C, the supernatant was collected and protein concentration was measured using a BCA Kit (Biosharp, Hefei, Anhui, China). Protein (40 µg) was separated by SDS–PAGE and electrically transferred to polyvinylidene fluoride membrane. Specific antibodies are used to detect the blots. Antibodies and dilution ratio of relevant proteins are given in Supplementary Table 4. All of the complete, unedited blots are exhibited in the supplemental material.

### Cell culture

Five PC cell lines (MIA PaCa-2, SW1990, CFPAC-1, PANC-1, and BxPC-3) and HPNE cells were acquired from The American Type Culture Collection. CFPAC-1 and SW1990 cells were cultured in IMEM (Gibco, Waltham, MA, USA), PANC-1 and MIA-PaCa2 cells were cultivated in DMEM (Gibco), HPNE and BXPC-3 cells were maintained in RPMI-1640 (Gibco). All cells were grown in a medium containing 10% fetal bovine serum (FBS; Gibco) and under a humid environment at 37 °C and 5% CO_2_. The mycoplasma-free status was confirmed by STR profiling.

### Cell infection and transfection

Short hairpin RNAs (shRNAs) that target FOXQ1 and the control lentivirus were purchased from Genechem (Shanghai, China). Small interfering RNAs (siRNAs) targeting LDHA were purchased from RiboBio (Guangzhou, China). FOXQ1-overexpressing plasmids and their corresponding control plasmids were obtained from WZ Biosciences, Inc. (Shandong, China). siRNA and plasmid transfection were performed using Lipofectamine 3000 (Invitrogen, Waltham, MA, USA). For the target sequences of sh-RNAs and si-RNAs see Supplementary Table 5.

### Cell counting Kit-8 assay

Different groups of PC cells were seeded in 96-well plates and cultured for 0, 24, 48, and 72 h (4 × 10^3^ cells/well, with six replicate wells per group). Next, 10 µl of CCK-8 solution (Dojindo Molecular Technologies, Inc., Japan) was added to each well at specified intervals. Absorbance was assessed at 450 nm after 2 h of culture. Three independent replicates were examined.

### Sphere formation assay

PC cells (2 × 10^3^/well) were cultured in DMEM-F12 with B27 (20 μl/ml), b-FGF (20 ng/ml), EGF (20 ng/ml), and 1% penicillin–streptomycin for 10–14 days. Cell spheroids were analyzed through a microscope (Olympus, Japan).

### EdU assay

On the day before the experiment, PC cells (5 × 10^5^ cells/well) were seeded in 24-well plates and then incubated with 10 μM EdU solution (Beyotime, Cat No. C0071S) for 2 h. Cells were fixed with 4% paraformaldehyde and washed with PBS, then permeabilized with 0.3% Triton X-100 and stained using Hoechst and Apollo reaction mixtures.

### Colony formation assay

The PC cells were inoculated in six-well plates for 14 days (1 × 10^3^ cells/well). Colonies were fixed with 4% paraformaldehyde and then stained with 0.3% crystal violet for 30 min, respectively.

### Immunofluorescence assay

PC cells seeded in 24-well plates were cultured for 24 h. The cells were fixed with 4% paraformaldehyde and permeabilized with 0.3% Triton X-100. After blocking the cells with 5% BSA in Triton X-100 for 1 h, cells were incubated with primary antibody at 4 °C overnight. The cells were then incubated with a secondary antibody at room temperature for 1 h. Nuclei were stained with DAPI for 15 min, and a fluorescence microscope (Zeiss, Germany) was utilized for viewing the cells. Three independent replicates were examined, and five randomly selected microscope images (×200 magnification) per treatment were obtained.

### Wound healing assay

PC cells were plated in six-well plates (2 × 10^5^/well) When cell confluence reached 95%, a linear wound was created with a 200-µl pipette tip. The cells were then cultured for 48 h in serum-free medium. Images were obtained using an inverted microscope at 0 and 48 h.

### Migration and invasion assays

Migration and invasion experiments were conducted in Transwell chambers (LABSELECT, Cat No. 14341). Starvation-treated cells (4 × 10^4^/well) were plated in the upper chamber and 700 µl complete medium was added to the lower chamber. 28 h later, cells in the upper chamber were removed, and the remaining cells were fixed and stained with 4% paraformaldehyde and 0.3% crystal violet, respectively. For invasion assay, 25 µl Matrigel (BD, Cat No. 3422356234) was applied for coating the upper membrane. Three independent replicates were examined, and five randomly selected microscope images (×200 magnification) per treatment were recorded.

### In vivo xenografts and metastasis models

Female six-week-old BALB/c nude mice (HFK Bio-Technology Co., Ltd, Beijing, China) were distributed at random to each group (the number of each group is 7). 2 × 10^6^ cells were injected into the right axilla of the mice for the xenograft model. Tumor volume was measured every week for the next five weeks following injection, and then mice were euthanized. The equation (length × width^2^)/2 was used to calculate the tumor volume. 1 × 10^6^ cells were injected into the spleen for the metastasis model (the number of each group is 5). 6 weeks later, the liver tissues were harvested and analyzed. The Guizhou Medical University Animal Care Welfare Committee approved all animal experiments.

### Oxygen consumption rate, extracellular acidification rate, glucose uptake, ATP production rate, and lactic acid production assays

PC cells (3.5 × 10^4^ cells/well) were seeded on XF96 microplates (Seahorse, Cat. No. 101085-004). The cell Mito Stress Test Kit (Cat No.103015-100) and Glycolysis Stress Test Kit (Seahorse, Cat No.103020-100) were adopted to detect the oxygen consumption rate and extracellular acidification rate by the Seahorse XFe96 Analyzer. Glucose uptake analyzed by the Seahorse XFe24 analyzer, ATP and lactate production were measured using the ATP Assay Kit (Sigma, MAK190) and Lactate Assay Kit (Sigma, MAK064).

### RNA-seq

Three pairs of FOXQ1 knockdown and corresponding negative control PANC-1 cell samples were applied to perform RNA-seq. Total RNA was extracted from each sample using the Novaseq 6000 platform. DEGs were defined using the following criteria: |log2FC | >1 and *p* < 0.05.

### ChIP

The SimpleChIP® Plus Sonication Chromatin IP Kit (Cell Signaling Technologies, Cat No. #56383) was exploited to conduct the ChIP assay. FOXQ1-overexpressing PANC-1 cells (1 × 10^7^) were fixed with 1% formaldehyde, the reaction was stopped by 0.1 M glycine. The chromatin was fragmented into sizes ranging from 200 to 1000 bp and then incubated with anti-Flag (Proteintech, Cat No. 20543-1-AP) or anti-IgG (Proteintech, Cat No. 30000-0-AP) antibody. DNA was amplified by RT-qPCR or utilized for ChIP-seq.

### Dual-luciferase reporter assay

PC cells were inoculated in 96-well plates (4 × 10^3^ cells/well). The luciferase reporter plasmid (150 ng) was then co-transfected with the pGL-4.74 Renilla control plasmid (Promega, catalog number E692A) (30 ng) using Lipofectamine 3000 (Invitrogen) reagent. 48 h later, luciferase activity was evaluated by the Dual-Luciferase Reporter Assay Kit (Promega, Madison, WI, USA). Three independent replicates were examined.

### Statistical analysis

The data were expressed as mean ± standard deviation and analyzed by SPSS (version 23.0; IBM Corp., Armonk, NY, USA). The two-sided unpaired Student’s *t*-test was applied for assessing the group differences. Survival was examined using Kaplan–Meier curves. Correlations between FOXQ1 and LDHA were identified by Pearson correlation analysis. *p* < 0.05 was considered of statistical significance.

## Results

### FOXQ1 is overexpressed in PC tissues and cells

**Fig. 1:**
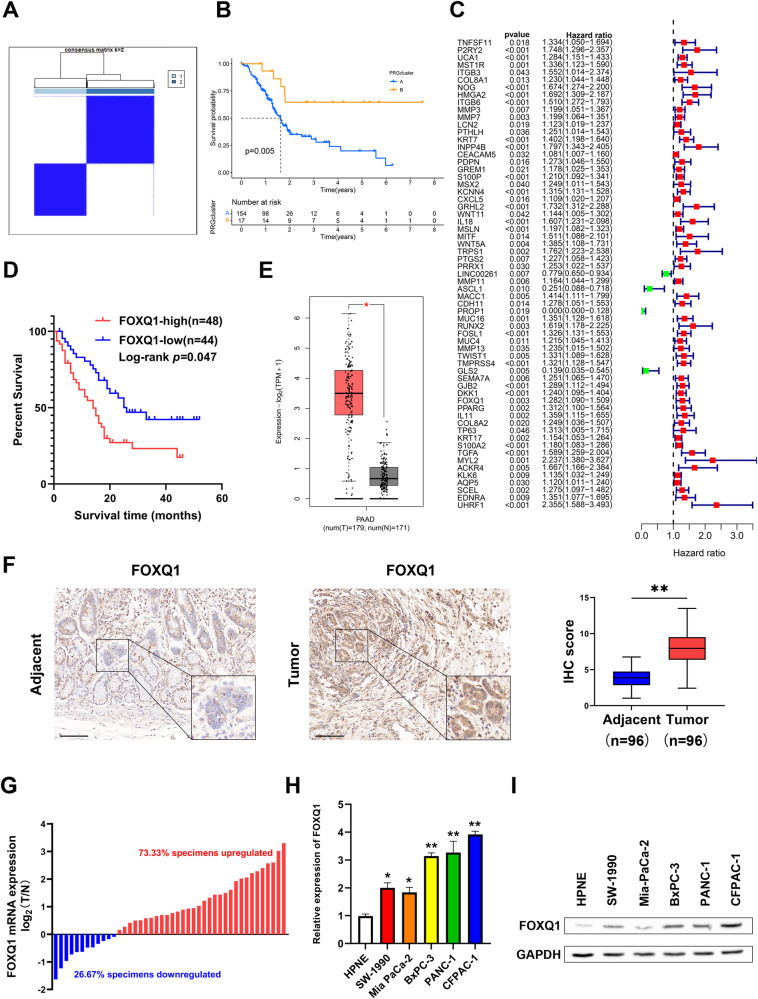
FOXQ1 is overexpressed in PC tissues and cells. Screening of PC tissues for the EMT-related differentially expressed transcription factor FOXQ1 by public databases. **A** Consensus matrix heatmap defining two clusters (*k* = 2) and their correlation area. **B** Kaplan–Meier curve of clusters A and B. **C** Mapping of forest plots; FOXQ1 was upregulated in PC samples and subgroup A (high risk) and associated with prognosis. **D** Kaplan–Meier curve of 92 PC patients on the basis of IHC scores. **E** FOXQ1 relative expression was examined from GEPIA databases. **F** FOXQ1 expression level was assessed in 96 pairs of PC and adjacent tissues using IHC. **G** RT-qPCR assay was used to measure the mRNA level of FOXQ1 in 45 pairs of PC and adjacent tissues. **H** RT-qPCR assay was used to measure the mRNA level of FOXQ1 in the indicated cells. **I** Western blotting analysis of FOXQ1 expression in the indicated cells. Scale bar: 100 µm; *p < 0.05; **p < 0.01.

We downloaded the data of RNA-seq of PC tissues from the TCGA database and screened these data with EMT-related dataset from the GESA database, and finally found that transcription factor FOXQ1 is a differentially expressed gene associated with EMT in PC tissues (Supplementary Fig. S1). Then we clustered the samples, gradually adding clustering variables (*k*) from 2 to 9. *k* = 2 was determined as the optimal number of clusters and the entire cohorts were divided into clusters A (high expression subgroup of EMT-related genes, *n* = 154) and B (low expression subgroup of EMT-related genes, *n* = 17) using a consensus clustering algorithm. By PCA analysis, we observed a significant difference in prognosis between PC patients in cluster A and cluster B (*P* < 0.01), with PC patients in cluster A having a significantly worse prognosis than those in cluster B (Fig. 1A, B). To further understand the EMT-related mechanisms, we analyzed the EMT-related genes with differential expression and prognostic relevance for clusters A and B and drew a forest plot (Fig. 1C). Moreover, PC patients with high FOXQ1 level showed shorter overall survival time (Fig. 1D). The mRNA level of FOXQ1 in 179 PC tissues and 171 normal pancreatic tissues was examined by GEPIA analysis, the finding demonstrated that PC tissues had greater level of FOXQ1 expression than normal pancreatic tissues (Fig. 1E). To validate the results from the bioinformatics study, we performed immunohistochemistry assay on 96 matched pairs of pancreatic tumor and surrounding non-tumor tissues. The results showed that PC tumor tissues had higher FOXQ1 levels than nearby non-tumor tissues (Fig. 1F). FOXQ1 mRNA was more abundant in 73.33% of PC samples in comparison with non-tumor samples (Fig. 1G). Further, we examined the expression of FOXQ1 in five human PC cell lines (SW1990, MIA PaCa-2, BxPC-3, PANC-1, and CFPAC-1) and the telomerase-immortalized human pancreatic duct derived (HPNE) cell. The results denoted that both the mRNA and protein levels of FOXQ1 were increased in PC cells than in the HPNE cell (Fig. 1H, I). These findings suggest that FOXQ1, an EMT-related target, is highly expressed in both PC tissues and cells.

### FOXQ1 facilitates PC proliferation and stemness in vitro and in vivo

**Fig. 2:**
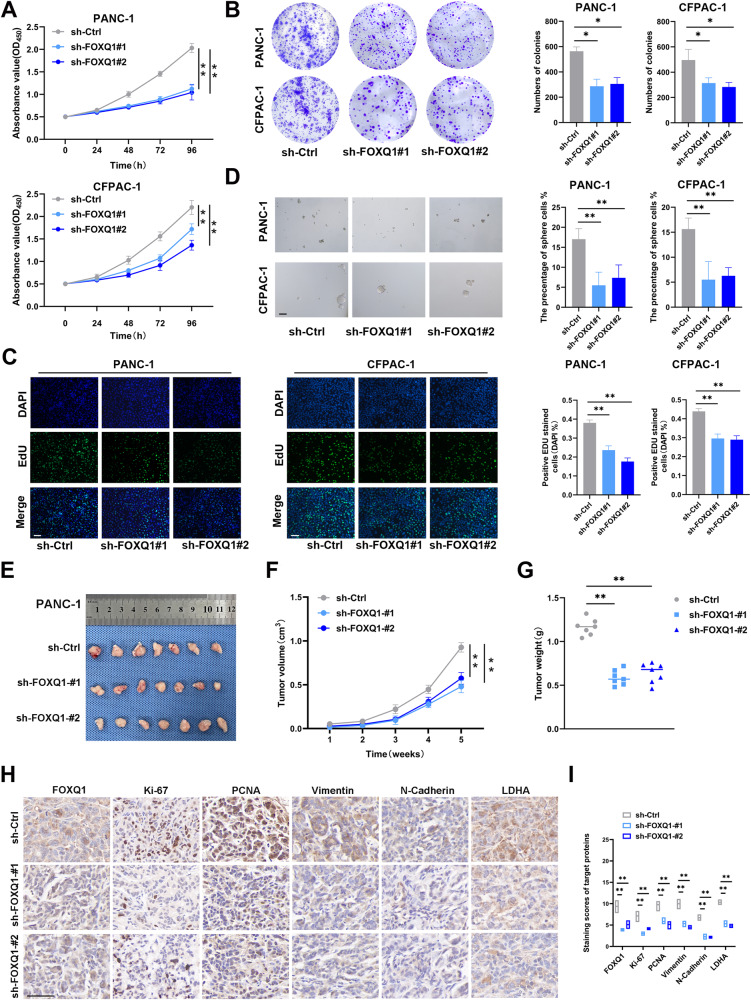
FOXQ1 promotes PC proliferation and stemness in vitro and in vivo. **A** CCK-8 evaluation of cell proliferation in the indicated cells. **B** Images of representative colonies of the indicated cells. **C** Images of representative PC cells stained with EdU. **D** Sphere formation assay of the indicated cells. **E** Subcutaneous tumors of the mice inoculated with the indicated cells. **F** Tumor growth curves. **G** Tumor weight. **H** IHC detection of FOXQ1, Ki-67, PCNA, Vimentin, N-Cadherin, and LDHA expression in subcutaneous tumors. **I** IHC score. Scale bar: 50 µm; *p < 0.05; **p < 0.01.

Stable FOXQ1 knockdown PC cell lines (PANC-1 and CFPAC-1 cells) were generated, and the sh-FOXQ1#1 and sh-FOXQ1#2 targets were chosen by RT-qPCR and western blot assays (Supplementary Fig. S2). The role of FOXQ1 in proliferation was examined by CCK-8, colony formation, and EdU staining assays. Knockdown of FOXQ1 decreased the capacity of cell proliferation in PANC-1 and CFPAC-1 cells (Fig. 2A–C). We also performed a suspension sphere-formation assay to determine FOXQ1’s contribution to tumor stemness and found that the FOXQ1 silencing groups exhibited decreased sphere formation efficiency (Fig. 2D).

To determine if FOXQ1 has contributed to the malignant activity of PC in vivo, we generated subcutaneous tumor nude mouse models. For the subcutaneous tumor model, mice were injected with control or FOXQ1-silenced PANC-1 cells. The results showed that mice injected with FOXQ1-silenced PANC-1 cells exhibited significantly smaller tumors compared with the control group mice (Fig. 2E–G). IHC assay revealed decreased expression of FOXQ1, Ki-67, PCNA, Vimentin, N-Cadherin, and LDHA in tumors from the FOXQ1-silenced group (Fig. 2H, I). All of the above implies that FOXQ1 may promote PC cell proliferation and stemness in vitro and in vivo.

### FOXQ1 promotes PC invasion and metastasis in vitro and in vivo

**Fig. 3:**
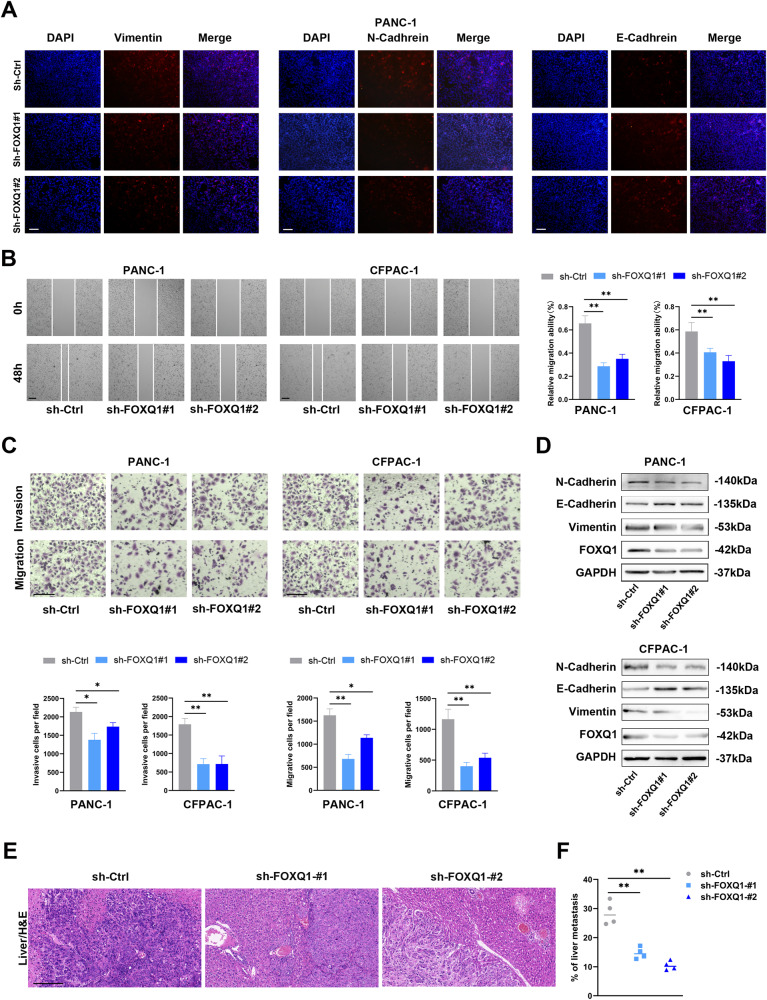
FOXQ1 accelerates PC cell invasion and metastasis in vitro and in vivo. **A** Immunofluorescence assay was conducted to evaluate the EMT-related protein level in PANC-1 cells. Scale bar: 100 µm. **B** Wound healing assay of PANC-1 and CFPAC-1 cell groups. Scale bar: 100 µm. **C** Representative images of the Transwell experiment in the indicated cells. Scale bar: 100 µm. **D** Western blot analysis of N-Cadherin, E-Cadherin, Vimentin, and FOXQ1 expression in the indicated cells. **E** Liver metastases. Scale bar: 500 µm. **F** Metastasis foci. *p < 0.05; **p < 0.01.

The capacity of FOXQ1 on PC invasion and metastasis was explored further. The result of the immunofluorescence assay demonstrated that the FOXQ1 knockdown group PC cells expressed a lower level of the mesenchymal-related proteins Vimentin and N-Cadherin and a higher level of the epithelial-related protein E-Cadherin compared with the control group (Fig. 3A and Supplementary Fig. S3). Moreover, wound healing and Transwell assays demonstrated that downregulating FOXQ1 decreased PC cell’s capacity for migration and invasion (Fig. 3B, C). The result of the western blot displayed analogous findings with immunofluorescence assay (Fig. 3D). To determine if FOXQ1 contributes to the malignant activity of PC in vivo, liver metastasis nude mouse models were generated. The results indicated that FOXQ1-silenced groups showed weaker metastasis ability compared with the control group (Fig. 3E, F). These findings suggest that FOXQ1 aggravates PC cell invasion and metastasis in vitro and in vivo.

### FOXQ1 activates metabolism-related pathways and promotes PC cell aerobic glycolysis in vitro

**Fig. 4:**
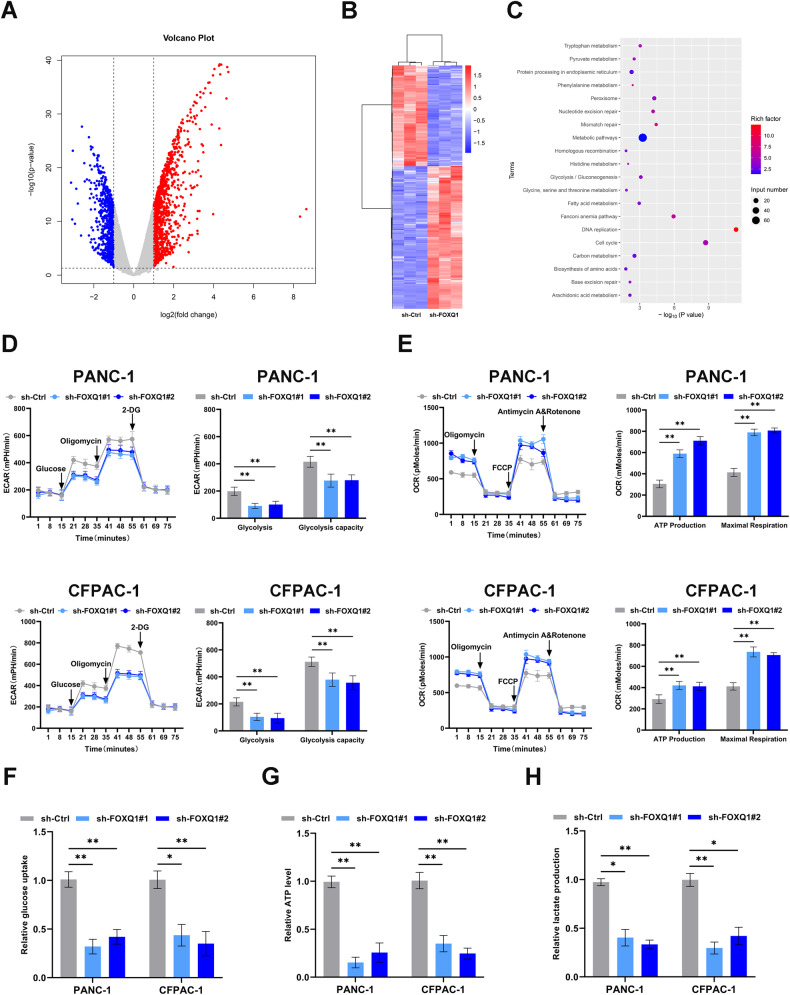
FOXQ1 activates metabolism-related pathways and promotes PC cell’s aerobic glycolysis in vitro. **A** Volcano map of the RNA-seq data. **B** Clustering heat map of the RNA-seq data. **C** Top 20 enriched KEGG pathway scatter plot. **D** ECAR assay of PANC-1 and CFPAC-1 cell groups. **E** The maximum respiration, ATP production, and oxygen consumption rates of PANC-1 and CFPAC-1 cell groups. **F–H** The relative glucose uptake (**F**), ATP level (**G**), and lactate production (**H**) in the indicated cells. *p < 0.05; **p < 0.01.

We next conducted RNA-seq to discern differentially expressed genes (DEGs) in FOXQ1-control and silenced PANC-1 cells using the |log2 FC | > 1 and *P* < 0.05 criteria (Fig. 4A, B). KEGG enrichment analysis revealed that the DEGs were enriched in the metabolic pathway (Fig. 4C). To support their powerful capability for invasion, proliferation, and metastasis, cancer cells convert from anaerobic to aerobic glycolysis. Thus, we extrapolated that FOXQ1 might be in relation to the regulation of aerobic glycolysis, which influences the prognosis of PC. The impact of FOXQ1 on PC cell glycolysis was investigated through the Seahorse XF extracellular flux analyzer. We discovered that FOXQ1 knockdown decreased glycolysis and glycolysis capacity (Fig. 4D), while enhanced ATP synthesis and maximum respiration (Fig. 4E) in PC cells. Furthermore, by detecting the cellular energy metabolism in PC cells we found that the glucose uptake, ATP production, and lactate production decreased after FOXQ1 knockdown (Fig. 4F–H). These results suggest that in vitro aerobic glycolysis and PC development may partially be increased by FOXQ1.

### FOXQ1 activates the transcription of LDHA

**Fig. 5:**
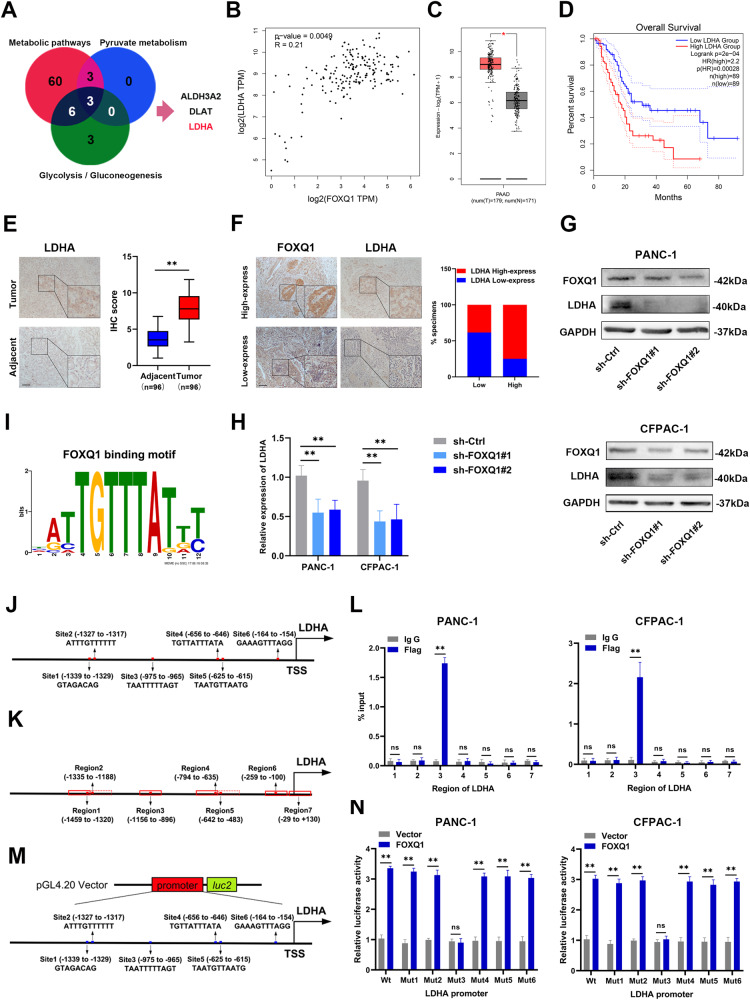
FOXQ1 activates the transcription of LDHA. **A** The intersection of pyruvate metabolism, glycolysis/gluconeogenesis, and differentially expressed genes (DEGs) in metabolic pathways is shown as a Venn diagram. **B** Correlation analysis of LDHA and FOXQ1 in PC using the GEPIA database. **C** Relative expression of LDHA in PC acquired from the GEPIA database. **D** Overall survival curve of PC patients with differential LDHA expression from the GEPIA database. **E** LDHA expression level was assessed in 96 pairs of PC (T) and corresponding adjacent (N) tissues by IHC assay. **F** Representative images of IHC assay for FOXQ1 and LDHA expression in successive slices of PC tissues. The expression of 40 samples was tested using Pearson’s chi-squared method. **G** and **H** Western blot (**G**) and RT-qPCR (**H**) analysis of LDHA expression in the indicated cells, respectively. **I** FOXQ1 binding motif predicted by TFDB. **J** Potential FOXQ1 binding locations in the LDHA promoter. **K** Diagrammatic representation of primers for LDHA areas. **L** Analysis of FOXQ1 enrichment on the LDHA promoter via ChIP-PCR. IgG served as the negative control. **M** Schematic of dual-luciferase reporter vectors. **N** Luciferase assays using the wild-type (Wt) and mutant (Mut) LDHA reporters in the indicated cells. Scale bar: 100 µm, *p < 0.05; **p < 0.01.

Based on the results of KEGG-enrichment analysis, three genes (including ALDH3A2, DLAT and LDHA) were identified after taking intersections in the sets of DEGs in metabolic pathways, pyruvate metabolism and glycolysis/gluconeogenesis pathways (Fig. 5A). The correlation of FOXQ1 with ALDH3A2, DLAT and LDHA in PC was analyzed using the GIEPA database, and the results showed that LDHA was most closely related to FOXQ1(*R* = 0.21, *p* = 0.0049) (Fig. 5B and Supplementary Fig. S4). The mRNA level of LDHA in 179 PC samples and 171 normal pancreatic tissues was investigated by GEPIA, and the result exhibited that the expression of LDHA was elevated in PC (Fig. 5C). In addition, PC patients with high LDHA levels presented shorter overall survival time (Fig. 5D). The protein expression level of FOXQ1 and LDHA were examined in PC samples by IHC assays. And the results denoted that PC tissues showed higher FOXQ1 and LDHA protein levels than that in the nearby non-tumor tissues (Fig. 5E). Moreover, FOXQ1 high-expressing specimens showed a significantly high level of LDHA expression (Fig. 5F). Western blotting and RT-qPCR results demonstrated that FOXQ1 knockdown attenuated LDHA expression in PC cells (Fig. 5G, H). We then matched the FOXQ1 binding sequences acquired from the TFDB database with the promoter region sequences of LDHA and discovered that the LDHA promoter may contain six FOXQ1 binding sites (Fig. 5I, J). With the probable transcriptional start site and binding sites included, PCR primers for about 160-bp region were designed (Fig. 5K). According to ChIP-PCR data, FOXQ1 could directly bind to the LDHA promoter’s regions (Fig. 5L). To clarify the transcriptional activity of FOXQ1, plasmids containing the promoter sequences of wild-type (Wt) and individual binding site mutants (Mut) were transfected into FOXQ1 overexpressing and control cells (Fig. 5M). Upregulation of FOXQ1 increased LDHA promoter activity, according to the dual-luciferase reporter assay. However, this effect was absent in the mutation of the FOXQ1 binding site (Fig. 5N). Together, these findings suggest that FOXQ1 could directly bind to the promoter region of LDHA to enhance its expression.

### LDHA is critical for FOXQ1-mediated PC progression

**Fig. 6:**
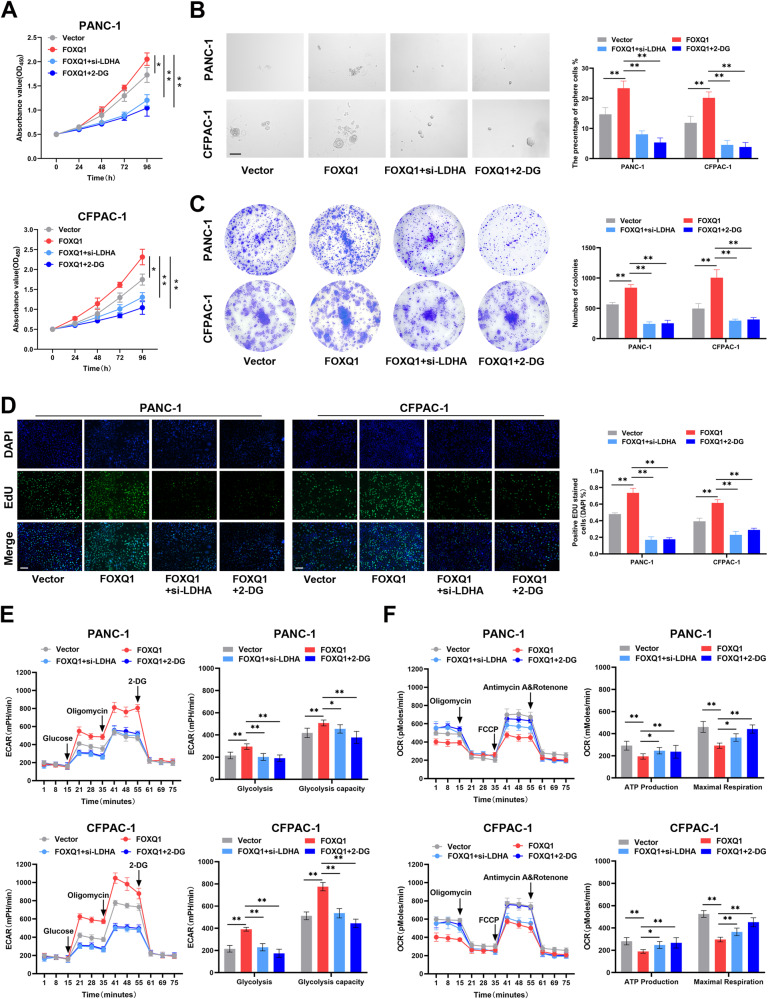
LDHA is essential for FOXQ1-mediated PC cell proliferation, stemness, and aerobic glycolysis in vitro. Small interfering RNA of LDHA (si-LDHA) and 2-DG (glycolytic pathway inhibitors) were added to PANC-1 and CFPAC-1 cells infected with lentivirus that overexpressed FOXQ1. **A** CCK-8 evaluation of cell proliferation of each group. **B** Sphere formation experiment. **C** Colony formation assay of each group. **D** EdU staining images of the indicated cell groups. **E** ECAR assay of the indicated cell groups. **F** OCR assay of the indicated cell groups. Scale bar: 100 µm; *p < 0.05; **p < 0.01.

**Fig. 7:**
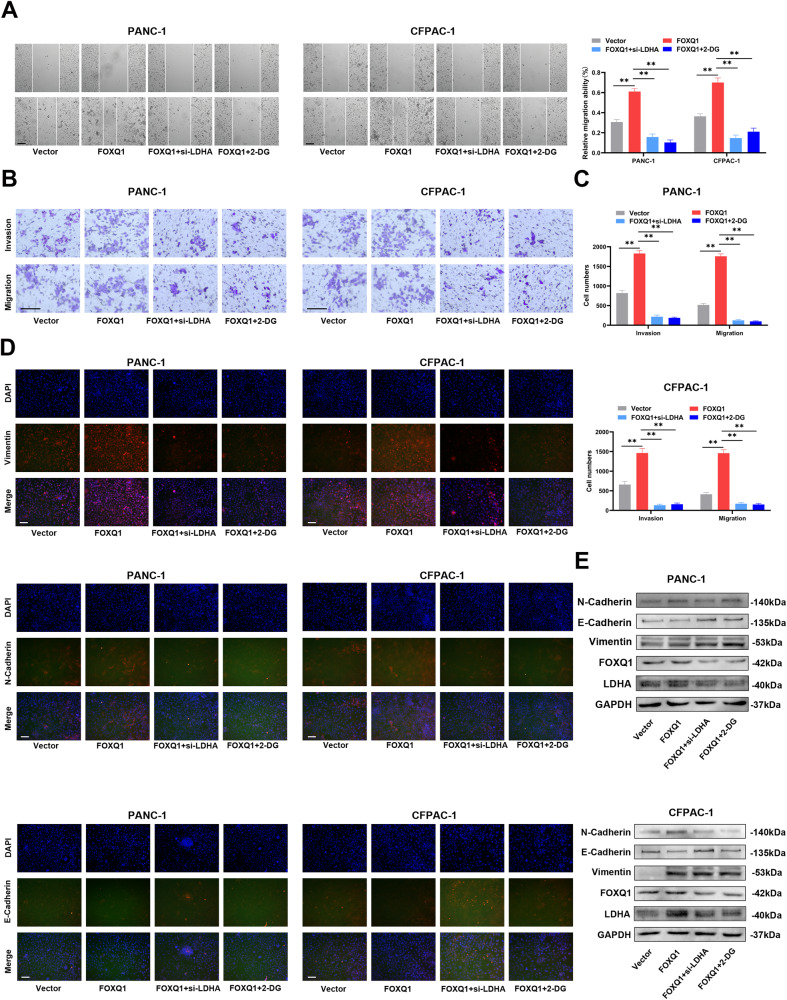
LDHA is essential for FOXQ1-mediated PC cell invasion and metastasis in vitro. Small interfering RNA of LDHA (si-LDHA) and 2-DG (glycolytic pathway inhibitors) were added to PANC-1 and CFPAC-1 cells infected with lentivirus that overexpressed FOXQ1. **A** Wound healing assay of PANC-1 and CFPAC-1 cell groups. **B**, **C** Representative images of the Transwell assay in the indicated cells. **D** Immunofluorescence assay was performed to detect the EMT-related protein expression in PANC-1 and CFPAC-1 cell groups. **E** Western blotting experiment to detect the level of N-Cadherin, E-Cadherin, Vimentin, FOXQ1, and LDHA. Scale bar: 100 µm; *p < 0.05; **p < 0.01.

In order to find out whether LDHA has a pivotal role in the PC progression mediated by FOXQ1, siRNA of LDHA (si-LDHA) and 2-deoxy-d-glucose (2-DG, the glycolysis inhibitors, 100 mM) were added into the FOXQ1 overexpressing PC cells. We found that inhibiting LDHA and glycolysis significantly reversed PC cell proliferation (Fig. 6A, C, D), stemness (Fig. 6B), invasion, and metastasis capacity augmented by FOXQ1 (Fig. 7A–C). Additionally, suppression of LDHA and glycolysis prevented the FOXQ1-enhanced extracellular acidification and oxygen consumption (Fig. 6E, F). Meanwhile, the effects of FOXQ1 on glucose uptake, ATP production, and lactate production in PC cells were restored by knocking down LDHA or the application of glycolysis inhibitors (Supplementary Fig. S5). Immunofluorescence (Fig. 7D) and western blotting analysis (Fig. 7E) confirmed that the level of Vimentin, N-Cadherin and E-Cadherin was partly reverted when downregulation of LDHA and glycolysis. Thus, it could be concluded that LDHA may be necessary for PC development mediated by FOXQ1.

## Discussion

PC is extremely aggressive, most PC patients show locally advanced disease (30–35%) or metastasis (50–55%) at the time of diagnosis [3, 26]. Despite the major advancements in PC prevention, detection, and therapy, efficient biomarkers and therapeutic approaches have not yet been discovered [7, 27]. Aerobic glycolysis (the Warburg effect) and EMT are significant contributors to the malignant activities of PC cells. A better understanding of the epithelial dynamics and metabolic state during metastasis is crucial for designing effective therapies for metastatic cancer including PC [28]. Aberrant activation of EMT, which is a critical mechanism in tissue repair and organ development [29], is a key factor in promoting distant metastasis and treatment resistance in cancer cells [30].

In this study, we screened the PC database and identified the TF FOXQ1, which is highly correlated with EMT, by bioinformatics analysis. FOXQ1 (namely HFH1), a member of the FOX gene family, is a sequence-specific TF [14]. A pan-cancer investigation revealed that the expression of FOXQ1 was connected to the activation and inactivation of 33 pathways in 12 tumors [13]. Many studies have shown a correlation between FOXQ1 and cancer stem cells. For example, miR-4319-mediated FOXQ1 inhibition suppressed EMT and prevented cancer stemness in hepatocellular carcinoma [31]. In PC, FOXQ1 overexpression promotes cancer stem cell resistance to radiotherapy [32].

We discovered that FOXQ1 is highly expressed in PC tissues and cell lines. Furthermore, there was a strong correlation between high FOXQ1 expression with poor clinical prognosis in PC patients. Additionally, our findings demonstrated that elevated FOXQ1 expression boosts PC cell proliferation, tumor stemness, invasion, and metastasis both in vitro and in vivo. Immunoblotting and immunofluorescence assays showed an expression correlation between FOXQ1 and the EMT-related proteins. Overall, our data support that FOXQ1 could be a valuable prognostic marker and therapeutic target in PC.

Unlike a large collection of solid tumors, PC tissue contains a significant amount of fibrotic inflammatory stroma, which fosters a hypoxic and nutrient-deficient environment for cancer cells [5]. PC cells have the capacity to undergo ‘metabolic reprogramming’ to aerobic glycolysis, and to meet their high energy needs and develop proliferation, tumor stemness, invasion, and metastasis activities [19, 33]. Thus, discovering novel molecular pathways of aerobic glycolysis in PC has enormous therapeutic significance for PC therapy.

Through RNA-seq, we discovered that FOXQ1 is strongly linked to metabolism-related pathways in PC cells, including pyruvate metabolism and glycolysis/gluconeogenesis metabolism. We also confirmed that low FOXQ1 expression inhibits aerobic glycolysis in PC cells. Furthermore, screening and validation assays revealed that FOXQ1 may activate the LDHA transcription by directly interacting with its promoter region. Nowadays, LDHA is reported to be an intracellular enzyme that completes the glycolytic cycle by reversibly converting pyruvate to lactate and NADH to NAD [34], it possesses the greatest affinity among the LDH family members to convert pyruvate to lactate [35]. The expression level of LDHA in PC tissues is correlated with clinicopathological characteristics: LDHA is overexpressed during pancreatic carcinogenesis and exhibits greater expression in more aggressive tumors [25]. Furthermore, there is a consistent relationship between high blood LDH levels and worse OS in patients with unresectable PDAC. A meta-analysis of 18 studies found that increased pretreatment serum lactate dehydrogenase values were related to poor OS [36]. In addition, serum LDH level is recognized as an independent and significant predictive factor following palliative treatment with gemcitabine in patients with advanced PDAC [37].

**Fig. 8:**
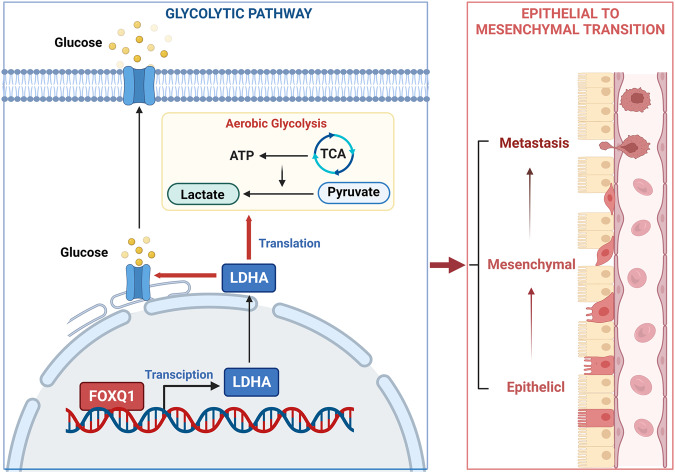
Schematic diagram of this study. An illustration of how FOXQ1 regulates LDHA-mediated aerobic glycolysis to promote PC cell proliferation, invasion, and metastasis was produced using BioRender.com (Agreement number: LZ25VCU6AY).

To sum up, our experimental studies show that FOXQ1 promotes the transcription of LDHA, then upregulates the level of aerobic glycolysis, thus facilitating PC cell proliferation, tumor stemness, invasion, and metastasis (Fig. 8). Therefore, FOXQ1 may be a possible therapeutic target in PC.

### Supplementary information

Supplementary figures and figure legends

Supplementary Table 1

Supplementary Tables(2-5)

Original Data File

Reproducibility checklist

## References

[1] Klein AP (2021). Pancreatic cancer epidemiology: understanding the role of lifestyle and inherited risk factors. Nat Rev Gastroenterol Hepatol.

[2] Veljkovikj I, Ilbawi AM, Roitberg F, Luciani S, Barango P, Corbex M (2022). Evolution of the joint International Atomic Energy Agency (IAEA), International Agency for Research on Cancer (IARC), and WHO cancer control assessments (imPACT Reviews). Lancet Oncol.

[3] Park W, Chawla A, O’Reilly EM (2021). Pancreatic cancer: a review. JAMA.

[4] Hingorani SR (2023). Epithelial and stromal co-evolution and complicity in pancreatic cancer. Nat Rev Cancer.

[5] Ren B, Cui M, Yang G, Wang H, Feng M, You L (2018). Tumor microenvironment participates in metastasis of pancreatic cancer. Mol Cancer.

[6] Zhou P, Li B, Liu F, Zhang M, Wang Q, Liu Y (2017). The epithelial to mesenchymal transition (EMT) and cancer stem cells: implication for treatment resistance in pancreatic cancer. Mol Cancer.

[7] Wood LD, Canto MI, Jaffee EM, Simeone DM (2022). Pancreatic cancer: pathogenesis, screening, diagnosis, and treatment. Gastroenterology.

[8] Herman L, Todeschini A-L, Veitia RA (2021). Forkhead transcription factors in health and disease. Trends Genet.

[9] Wang P, Lv C, Zhang T, Liu J, Yang J, Guan F (2017). FOXQ1 regulates senescence-associated inflammation via activation of SIRT1 expression. Cell Death Dis.

[10] Cui Y, Qiao A, Jiao T, Zhang H, Xue Y, Zou Y (2016). The hepatic FOXQ1 transcription factor regulates glucose metabolism in mice. Diabetologia.

[11] Yang Y, Ma Y, Li M, Zhu H, Shi P, An R (2023). STUB1 directs FOXQ1-mediated transactivation of Ldha gene and facilitates lactate production in mouse Sertoli cells. Cell Tissue Res.

[12] Zhu H, Ji H, Chen W, Han L, Yu L (2022). Integrin subunit β-like 1 mediates angiotensin II-induced myocardial fibrosis by regulating the forkhead box Q1/Snail axis. Arch Biochem Biophys.

[13] Dong Q, Yan L, Xu Q, Hu X, Yang Y, Zhu R (2022). Pan-cancer analysis of forkhead box Q1 as a potential prognostic and immunological biomarker. Front Genet.

[14] Hong X, Liu N, Liang Y, He Q, Yang X, Lei Y (2020). Circular RNA CRIM1 functions as a ceRNA to promote nasopharyngeal carcinoma metastasis and docetaxel chemoresistance through upregulating FOXQ1. Mol Cancer.

[15] Mitchell AV, Wu L, James Block C, Zhang M, Hackett J, Craig DB (2022). FOXQ1 recruits the MLL complex to activate transcription of EMT and promote breast cancer metastasis. Nat Commun.

[16] Xiang D, Gu M, Liu J, Dong W, Yang Z, Wang K (2023). m6A RNA methylation-mediated upregulation of HLF promotes intrahepatic cholangiocarcinoma progression by regulating the FZD4/β-catenin signaling pathway. Cancer Lett.

[17] Yang M, Liu Q, Dai M, Peng R, Li X, Zuo W (2022). FOXQ1-mediated SIRT1 upregulation enhances stemness and radio-resistance of colorectal cancer cells and restores intestinal microbiota function by promoting β-catenin nuclear translocation. J Exp Clin Cancer Res.

[18] Zheng J, Dou R, Zhang X, Zhong B, Fang C, Xu Q (2023). LINC00543 promotes colorectal cancer metastasis by driving EMT and inducing the M2 polarization of tumor associated macrophages. J Transl Med.

[19] Qin C, Yang G, Yang J, Ren B, Wang H, Chen G (2020). Metabolism of pancreatic cancer: paving the way to better anticancer strategies. Mol Cancer.

[20] Cao L, Wu J, Qu X, Sheng J, Cui M, Liu S (2020). Glycometabolic rearrangements–aerobic glycolysis in pancreatic cancer: causes, characteristics and clinical applications. J Exp Clin Cancer Res.

[21] Feng Y, Xiong Y, Qiao T, Li X, Jia L, Han Y (2018). Lactate dehydrogenase A: a key player in carcinogenesis and potential target in cancer therapy. Cancer Med.

[22] Sharma D, Singh M, Rani R (2022). Role of LDH in tumor glycolysis: regulation of LDHA by small molecules for cancer therapeutics. Semin Cancer Biol.

[23] Prasad CP, Gogia A, Batra A (2022). Essential role of aerobic glycolysis in epithelial-to-mesenchymal transition during carcinogenesis. Clin Transl Oncol.

[24] Pouysségur J, Marchiq I, Parks SK, Durivault J, Ždralević M, Vucetic M (2022). ‘Warburg effect’ controls tumor growth, bacterial, viral infections and immunity—genetic deconstruction and therapeutic perspectives. Semin Cancer Biol.

[25] Comandatore A, Franczak M, Smolenski RT, Morelli L, Peters GJ, Giovannetti E (2022). Lactate dehydrogenase and its clinical significance in pancreatic and thoracic cancers. Semin Cancer Biol.

[26] Cai B, Zhang K, Miao Y (2022). Incidence of pancreatic cancer by age and sex in the US from 2000 to 2018. JAMA.

[27] Sherman MH, Beatty GL (2023). Tumor microenvironment in pancreatic cancer pathogenesis and therapeutic resistance. Annu Rev Pathol.

[28] Fedele M, Sgarra R, Battista S, Cerchia L, Manfioletti G (2022). The epithelial-mesenchymal transition at the crossroads between metabolism and tumor progression. Int J Mol Sci.

[29] Dongre A, Weinberg RA (2019). New insights into the mechanisms of epithelial-mesenchymal transition and implications for cancer. Nat Rev Mol Cell Biol.

[30] Lu W, Kang Y (2019). Epithelial–mesenchymal plasticity in cancer progression and metastasis. Dev Cell.

[31] Han S, Shi Y, Sun L, Liu Z, Song T, Liu Q (2019). MiR-4319 induced an inhibition of epithelial-mesenchymal transition and prevented cancer stemness of HCC through targeting FOXQ1. Int J Biol Sci.

[32] Bao B, Azmi AS, Aboukameel A, Ahmad A, Bolling-Fischer A, Sethi S (2014). Pancreatic cancer stem-like cells display aggressive behavior mediated via activation of FoxQ1. J Biol Chem.

[33] Yang J, Ren B, Yang G, Wang H, Chen G, You L (2020). The enhancement of glycolysis regulates pancreatic cancer metastasis. Cell Mol Life Sci.

[34] Khan AA, Allemailem KS, Alhumaydhi FA, Gowder SJT, Rahmani AH (2020). The biochemical and clinical perspectives of lactate dehydrogenase: an enzyme of active metabolism. Endocr Metab Immune Disord Drug Targets.

[35] Li Z, Zhang H (2016). Reprogramming of glucose, fatty acid and amino acid metabolism for cancer progression. Cell Mol Life Sci.

[36] Gan J, Wang W, Yang Z, Pan J, Zheng L, Yin L (2018). Prognostic value of pretreatment serum lactate dehydrogenase level in pancreatic cancer patients: a meta-analysis of 18 observational studies. Medicine (Baltimore).

[37] Yu S-L, Xu L-T, Qi Q, Geng Y-W, Chen H, Meng Z-Q (2017). Serum lactate dehydrogenase predicts prognosis and correlates with systemic inflammatory response in patients with advanced pancreatic cancer after gemcitabine-based chemotherapy. Sci Rep.

