# Supplementary material for: FOXQ1 promotes pancreatic cancer cell proliferation, tumor stemness, invasion and metastasis through regulation of LDHA-mediated aerobic glycolysis
Source: Cell Death Dis. 2023 Oct 24;14(10):699. doi: 10.1038/s41419-023-06207-y (PMC10598070; doi:10.1038/s41419-023-06207-y)
Supplement: Supplementary file 1 — Supplementary figures and figure legends [file 41419_2023_6207_MOESM1_ESM.pdf]

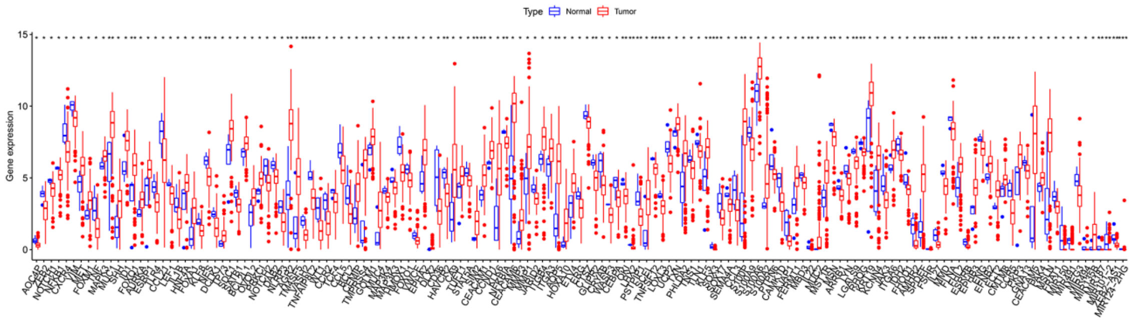

Supplementary Fig.S1: The result of bioinformatics prediction analysis.

Screening of PC tissues for the EMT-related differentially expressed transcription factor FOXQ1 by public databases. EMT-related gene sets were retrieved from the GSEA database and analyzed with TCGA database.

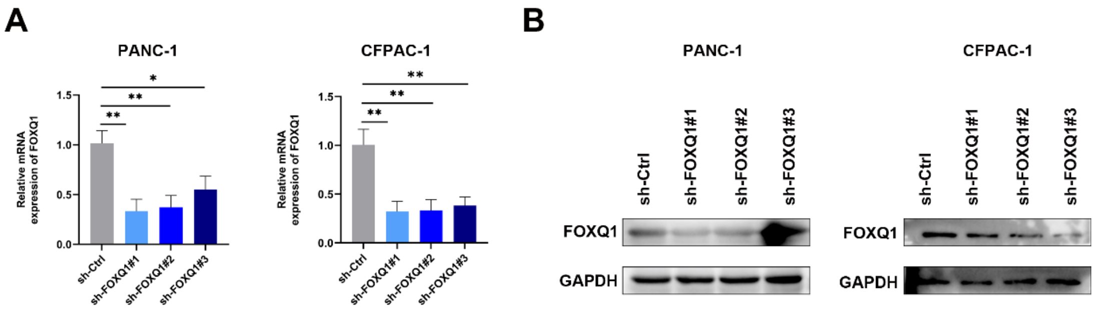

Supplementary Fig.S2: The expression confirmation of the stable FOXQ1 knockdown in the indicated cells.

A RT-qPCR analysis of FOXQ1 mRNA expression in PANC-1 and CFPAC-1 cells. **B** Western blot analysis of FOXQ1 expression in PANC-1 and CFPAC-1 cells.

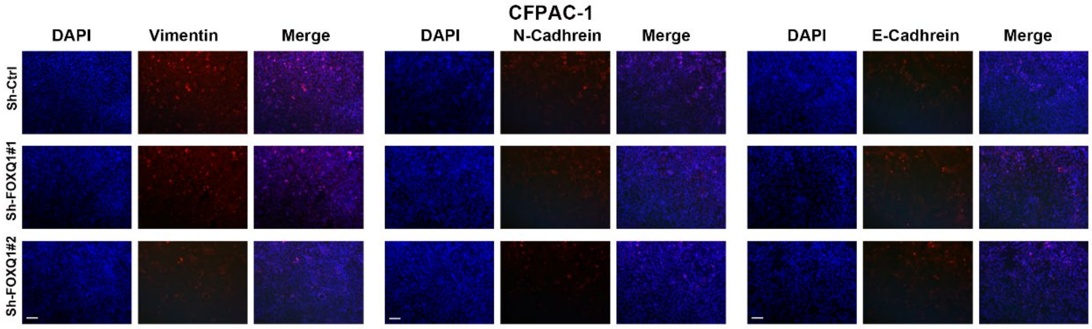

Supplementary Fig.S3: FOXQ1 promotes PC cells invasion and metastasis in vitro.

Immunofluorescence assay was performed to detect the expression of EMT-related proteins in CFPAC-1 cell. Scale bar: 100 μm; *$p < 0.05$; **$p < 0.01$.

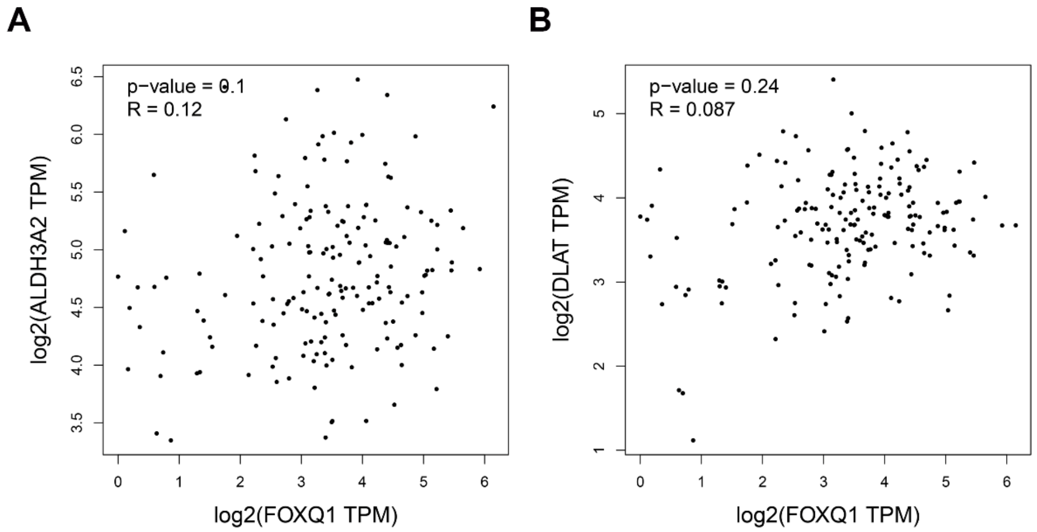

Supplementary Fig.S4: Correlation analysis of FOXQ1 with ALDH3A2 and DLAT.

A Correlation analysis of FOXQ1 and ALDH3A2 in PC using the GEPIA database. **B** Correlation analysis of FOXQ1 and DLAT in PC using the GEPIA database.

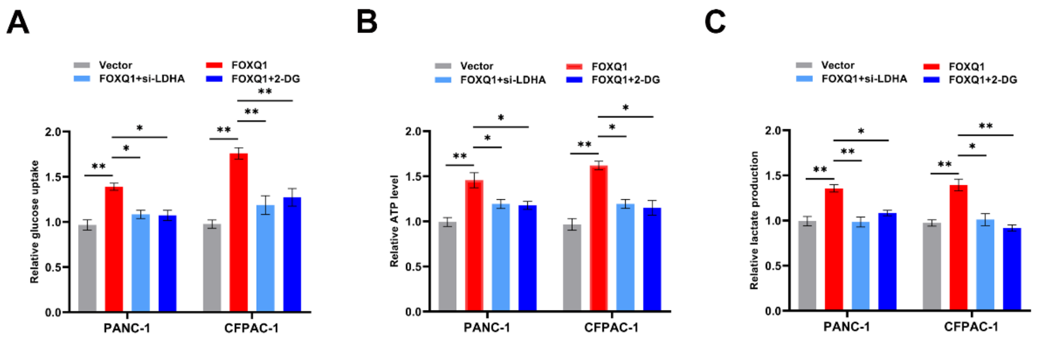

Supplementary Fig.S5: FOXQ1 accelerates the aerobic glycolysis through LDHA.

A-C. The relative glucose uptake (**A**), ATP level (**B**) and lactate production (**C**) in the indicated PC cells.
