# Supplementary material for: FOXQ1 promotes pancreatic cancer cell proliferation, tumor stemness, invasion and metastasis through regulation of LDHA-mediated aerobic glycolysis
Source: Cell Death Dis. 2023 Oct 24;14(10):699. doi: 10.1038/s41419-023-06207-y (PMC10598070; doi:10.1038/s41419-023-06207-y)
Supplement: Supplementary file 3 — Supplementary Tables(2-5) [file 41419_2023_6207_MOESM3_ESM.docx]

**Supplementary Table 2** Primer sequences of relevant genes used.

| **Gene** | **Sequence（5′ ->3′）** | |
| --- | --- | --- |
| FOXQ1 | Forward Primer | CACGCAGCAAGCCATATACG |
|  | Reverse Primer | CGTTGAGCGAAAGGTTGTGG |
| GAPDH | Forward Primer | CTCCAAAATCAAGTGGGGCG |
|  | Reverse Primer | TGGTTCACACCCATGACGAA |
| LDHA | Forward Primer | ACTCCATACAGGCACACTGG |
|  | Reverse Primer | TCTGGCAAAGTGGATATCTTGA |

**Supplementary Table 3** Primer sequences of ChIP-PCR.

| **Promoter** | **Region** | **Primers** | **Sequence( 5′ to 3′)** |
| --- | --- | --- | --- |
| LDHA | 1 | Forward primer | TCCAGAAGCACAGCCCAGAG |
|  |  | Reverse primer | CTTGTTCCGCATCCACCC |
|  | 2 | Forward primer | TAACAAATAGGGATTCCGAAAG |
|  |  | Reverse primer | TGGGAGTTCATGGGATGA |
|  | 3 | Forward primer | AGGCTTTGGTGGGAGGAT |
|  |  | Reverse primer | GAGGTGCAATTTCCAGATAA |
|  | 4 | Forward primer | GGATTCGGAGGTTACAGT |
|  |  | Reverse primer | ATCCTGGGCGAGAGCCTG |
|  | 5 | Forward primer | CAGGAACTCGGGAACAGGAT |
|  |  | Reverse primer | GCATACCCATCACAAACC |
|  | 6 | Forward primer | GGACACAGGGAAGGGAGA |
|  |  | Reverse primer | CCACGTAGGGTGTAGAAAGA |
|  | 7 | Forward primer | CTGGGTCCTATAAACGCTACG |
|  |  | Reverse primer | GGACTCCCACTGCGACTCTGG |

**Supplementary Table 4** Antibodies and dilution ratio of relevant proteins used.

| **Gene** | **Brand** | **Catalog number** | **WB** | **IP** | **IHC** | **IF** |
| --- | --- | --- | --- | --- | --- | --- |
| FOXQ1 | Proteintech | 23718-1-AP | 1:1000 | 1:50 | 1:100 | 1:200 |
| LDHA | Proteintech | 66287-1-Ig | 1:1000 | 1:50 | 1:100 |  |
| Flag | Proteintech | 20543-1-AP |  | 1:50 |  |  |
| IgG | Proteintech | 30000-0-AP |  | 1:50 |  |  |
| Ki-67 | Servicebio | GB111499 |  |  | 1:500 |  |
| PCNA | Servicebio | GB11010 |  |  | 1:500 |  |
| GAPDH | Cell Signaling | 5174 | 1:1000 |  |  |  |
| E-Cadherin | Cell Signaling | 14472 | 1:1000 |  | 1:100 | 1:100 |
| N-Cadhrein | Cell Signaling | 13116 | 1:1000 |  | 1:100 | 1:100 |
| Vimtenin | Signalway | 33541 | 1:1000 |  | 1:100 | 1:200 |

**Supplementary Table 5** Target sequences of relevant genes used.

| **Gene** | **Targeting sequences** |
| --- | --- |
| sh-FOXQ1#1 | 5′-GCTGGCCAACTATGAATTTGA-3′ |
| sh-FOXQ1#2 | 5′-GCATTGATCTGGAGAACATTG-3′ |
| si-LHDA | 5′- GAGGTCCTCTGCATGGATT-3′ |
