# Supplementary figures and images for: FOXQ1 promotes pancreatic cancer cell proliferation, tumor stemness, invasion and metastasis through regulation of LDHA-mediated aerobic glycolysis
Source: Cell Death Dis. 2023 Oct 24;14(10):699. doi: 10.1038/s41419-023-06207-y (PMC10598070; doi:10.1038/s41419-023-06207-y)

Supplemental Material – Original Blots


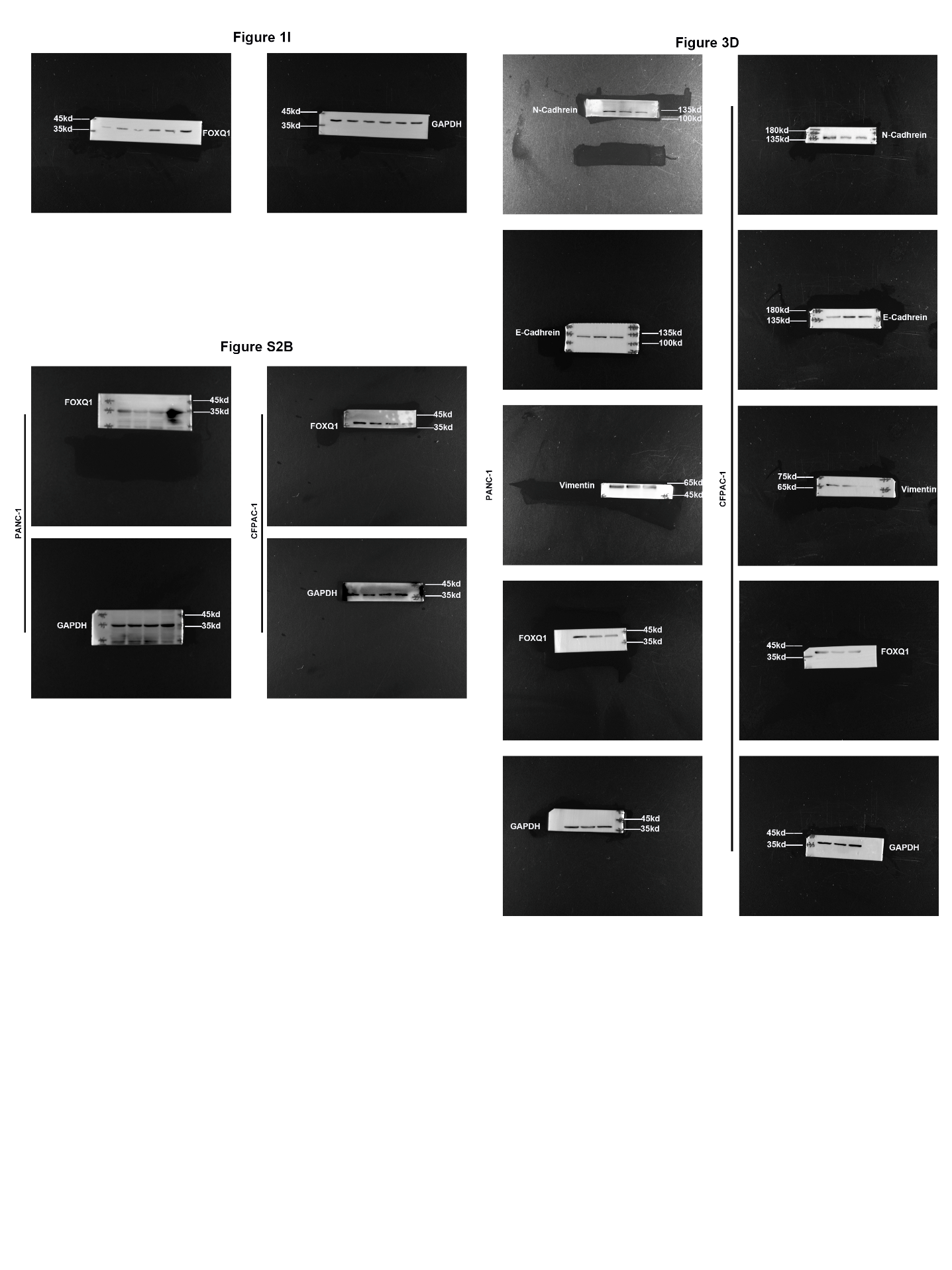


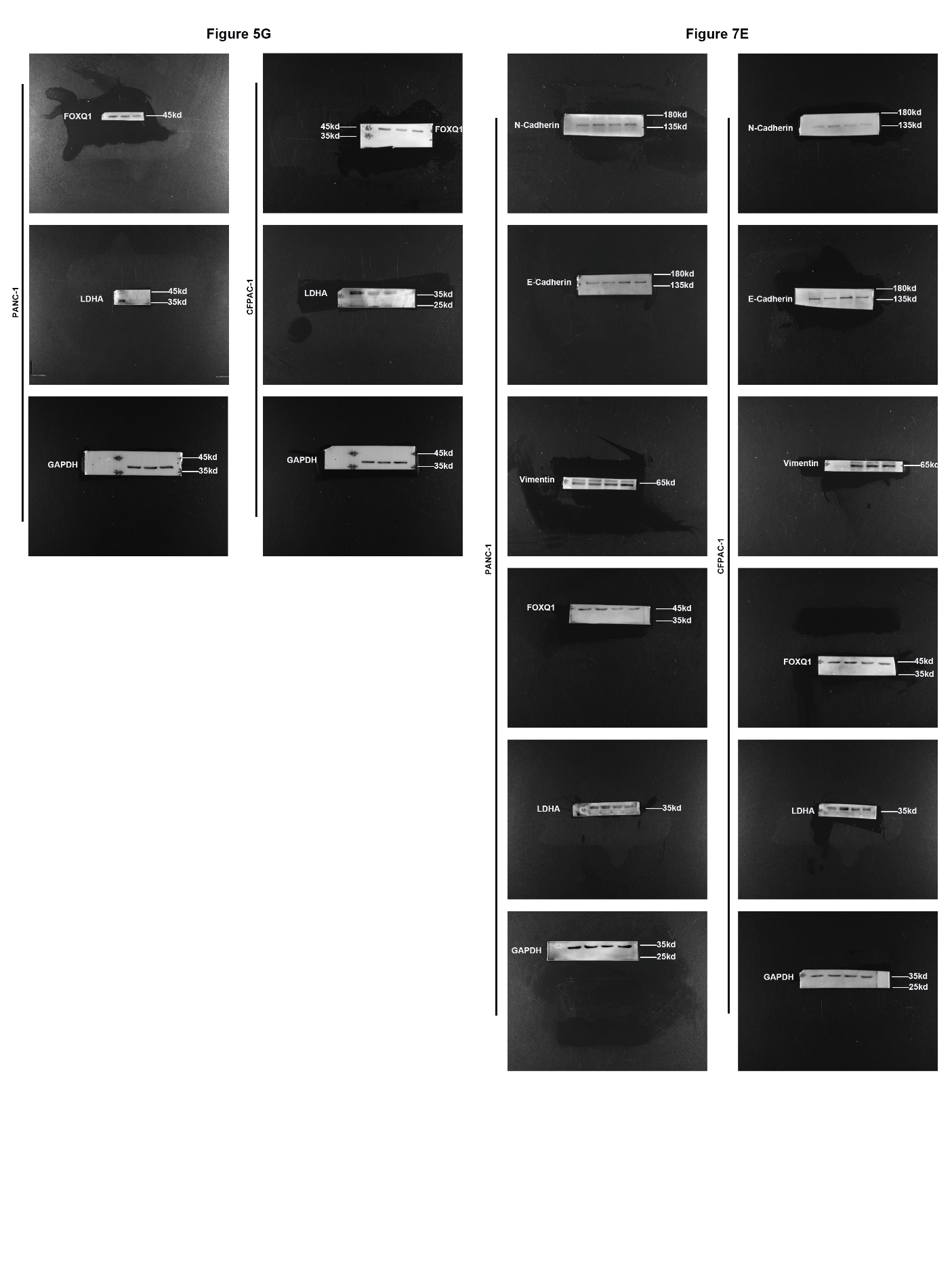

Supplement: Supplementary file 4 — Original Data File [file 41419_2023_6207_MOESM4_ESM.docx]
